# Implementation of Case-Based Surveillance and Real-time Polymerase Chain Reaction to Monitor Bacterial Meningitis Pathogens in Chad

**DOI:** 10.1093/infdis/jiz366

**Published:** 2019-10-31

**Authors:** Marietou F Paye, Kadidja Gamougame, Sarah K Payamps, Alicia R Feagins, Daugla Doumagoum Moto, Ronelngar Moyengar, Nathan Naïbeï, Jeni Vuong, Alpha Oumar Diallo, Ashley Tate, Heidi M Soeters, Xin Wang, Mahamat Ali Acyl

**Affiliations:** 1 Centers for Disease Control and Prevention Foundation; 2 IHRC Inc; 3 National Center for Immunization and Respiratory Diseases, Centers for Disease Control and Prevention, Atlanta, Georgia; 4 Hôpital Général de Référence Nationale, N’Djamena, Chad; 5 Centre de Support en Santé Internationale, N’Djamena, Chad; 6 Service de Surveillance Épidémiologique Intégrée, Ministère de la Santé Publique, N’Djamena, Chad

**Keywords:** Chad, rt-PCR, MenAfriNet, bacterial meningitis, sub-Saharan Africa, culture, latex

## Abstract

**Background:**

Meningococcal serogroup A conjugate vaccine (MACV) was introduced in Chad during 2011–2012. Meningitis surveillance has been conducted nationwide since 2003, with case-based surveillance (CBS) in select districts from 2012. In 2016, the MenAfriNet consortium supported Chad to implement CBS in 4 additional districts and real-time polymerase chain reaction (rt-PCR) at the national reference laboratory (NRL) to improve pathogen detection. We describe analysis of bacterial meningitis cases during 3 periods: pre-MACV (2010–2012), pre-MenAfriNet (2013–2015), and post-MenAfriNet (2016–2018).

**Methods:**

National surveillance targeted meningitis cases caused by *Neisseria meningitidis*, *Haemophilus influenzae*, and *Streptococcus pneumoniae*. Cerebrospinal fluid specimens, inoculated trans-isolate media, and/or isolates from suspected meningitis cases were tested via culture, latex, and/or rt-PCR; confirmed bacterial meningitis was defined by a positive result on any test. We calculated proportion of suspected cases with a specimen received by period, and proportion of specimens with a bacterial meningitis pathogen identified, by period, pathogen, and test.

**Results:**

The NRL received specimens for 6.8% (876/12813), 46.4% (316/681), and 79.1% (787/995) of suspected meningitis cases in 2010–2012, 2013–2015, and 2016–2018, respectively, with a bacterial meningitis pathogen detected in 33.6% (294/876), 27.8% (88/316), and 33.2% (261/787) of tested specimens. The number of *N. meningitidis* serogroup A (NmA) among confirmed bacterial meningitis cases decreased from 254 (86.4%) during 2010–2012 to 2 (2.3%) during 2013–2015, with zero NmA cases detected after 2014. In contrast, proportional and absolute increases were seen between 2010–2012, 2013–2015, and 2016–2018 in cases caused by *S. pneumoniae* (5.1% [15/294], 65.9% [58/88], and 52.1% [136/261]), NmX (0.7% [2/294], 1.1% [1/88], and 22.2% [58/261]), and Hib (0.3% [1/294], 11.4% [10/88], and 14.9% [39/261]). Of specimens received at the NRL, proportions tested during the 3 periods were 47.7% (418), 53.2% (168), and 9.0% (71) by latex; 81.4% (713), 98.4% (311), and 93.9% (739) by culture; and 0.0% (0), 0.0% (0), and 90.5% (712) by rt-PCR, respectively. During the post-MenAfriNet period (2016–2018), 86.1% (678) of confirmed cases were tested by both culture and rt-PCR, with 12.5% (85) and 32.4% (220) positive by culture and rt-PCR, respectively.

**Conclusions:**

CBS implementation was associated with increased specimen referral. Increased detection of non-NmA cases could reflect changes in incidence or increased sensitivity of case detection with rt-PCR. Continued surveillance with the use of rt-PCR to monitor changing epidemiology could inform the development of effective vaccination strategies.

For more than a century, a region of sub-Saharan Africa known as the meningitis belt has seen large-scale epidemics of bacterial meningitis [1]. Historically, most epidemics were caused by *Neisseria meningitidis* serogroup A (NmA); however, outbreaks of serogroup C, X, and W (NmC, NmX, and NmW, respectively) have also occurred in the region [2, 3]. Chad, a landlocked country in north-central Africa with its southern region falling within the meningitis belt, has experienced meningitis outbreaks reported since 1916 [1]. Major epidemics occurred in 1924 and from 1935 to 1939, with mortality rates over 75% [4]. Epidemics happened periodically over the next 3 decades [4]. In response, there have been meningococcal polysaccharide vaccination campaigns to control outbreaks. In the absence of routine vaccination, outbreaks continued to occur in the 1980s and 2000s [5]. From 2009 to 2012, Chad experienced a nationwide NmA epidemic with nearly 15 000 suspected meningitis cases reported [3].

An affordable meningococcal serogroup A conjugate vaccine (MACV, MenAfriVac) was developed specifically for the meningitis belt. Vaccine rollout in the region between 2010 and 2018 was highly successful, with over 300 million people vaccinated in 22 of 26 countries in the meningitis belt [6, 7]. MACV rollout targeted individuals aged 1–29 years through mass vaccination campaigns with the goal of covering at least 90% of the target population. In Chad, the vaccine was introduced to N’Djamena, Mayo Kebbi Est, and Chari Baguirmi regions in 2011 and was later expanded to the rest of the country in 2012 [8].

With MACV introduction, it was important to assess vaccine impact on disease incidence and asymptomatic nasopharyngeal carriage of *N. meningitidis* [9]. The African Meningococcal Carriage Consortium (MenAfriCar) was established in 2009 to measure the prevalence of meningococcal carriage before and after the introduction of MACV, and included Chad, Ethiopia, Ghana, Mali, Niger, Nigeria, and Senegal [10]. MACV was effective at decreasing NmA carriage prevalence among individuals aged 1–29 years from 0.7% before the MACV vaccination campaign to 0.02% after vaccination [4]. The MenAfriNet consortium was established in 2014 to monitor the impact of MACV on disease burden through strengthening case-based surveillance (CBS) for meningitis [11]. Burkina Faso, Niger, and Togo joined MenAfriNet in 2014, followed by Mali in 2015 and Chad in 2016. MenAfriNet also supported the strengthening of country laboratory systems for the diagnosis of bacterial meningitis through the implementation of real-time polymerase chain reaction (rt-PCR).

MenAfriNet’s strategy allowed for timely monitoring of epidemiological changes through active and rapid species detection, as well as *N. meningitidis* capsular genogrouping and *Haemophilus influenzae* capsular genotyping for serotype b. This report describes current bacterial meningitis epidemiology in Chad and evaluates the impact of MenAfriNet-supported CBS and rt-PCR on specimen referral to the national reference laboratory (NRL) and pathogen confirmation, respectively.

## METHODS

### Bacterial Meningitis Surveillance

Since 2003, Chad has collected enhanced meningitis surveillance (EMS) data nationwide. For EMS, aggregate case counts of suspected meningitis, along with some basic demographic information, are collected through a district-level line list. Cerebrospinal fluid (CSF) specimens are collected from a subset of patients, with confirmatory testing conducted on specimens from the first 30 cases during epidemics [12, 13].

Starting in 2012, select districts implemented CBS during different time periods. Under CBS, the collection of clinical and demographic information, as well as CSF specimens, is required from all suspected cases of meningitis. From March 2012 through June 2013, MenAfriCar supported CBS in 12 districts (4 districts in each of 3 regions: Chari Baguirmi, Mayo Kebbi Est, and N’Djamena) after MACV introduction in these 3 regions [14]. CBS surveillance began in the Moïssala district (Mandoul region) in 2012 with the support of Médecins Sans Frontières (MSF) [15]. Starting in 2016, MenAfriNet supported CBS in 4 additional districts (Goundi, Mani, and Bedjondo, all 3 in the Mandoul region; and Massakory of the Hadjer-Lamis region) and implemented rt-PCR at the NRL [6, 16].

For both EMS and CBS, specimens from suspected meningitis cases reported from all districts in the country are transported to the NRL for testing. Meningitis case definitions follow World Health Organization (WHO) guidelines for enhanced meningitis surveillance in the African meningitis belt [14]. A suspected meningitis case is defined as sudden onset of fever above 38.5°C (rectal) or 38.0°C (axillary) and at least 1 of the following symptoms: stiff neck, altered consciousness, or other meningeal signs [7, 13]. A case is confirmed if 1 of the main causative meningitis pathogens (*N. meningitidis*, *H. influenzae*, or *Streptococcus pneumoniae*) is detected by culture, rt-PCR, and/or latex agglutination [7]. Nationwide surveillance data (EMS and CBS) were analyzed for this study.

### Laboratory Testing and Data Analysis

WHO guidelines recommend laboratories and hospitals collect 3–4 mL of CSF, of which 250 μL is aliquoted into a cryotube for PCR testing at the NRL, 500–1000 μL is inoculated into trans-isolate media for transport and culture-based testing at the NRL, and ≥1000 μL is aliquoted into a dry tube for probable case testing at the peripheral-level laboratories [17]. The NRL tested CSF specimens using the latex agglutination test by Pastorex (latex; Bio-Rad Laboratories, France; for the identification of NmA, NmB/*Escherichia coli*, NmC, NmY/W, *H. influenzae* serotype b [Hib], *S. pneumoniae*, and group B *Streptococcus*), following the manufacturer’s guidelines, and with CSF cultured on blood agar and/or chocolate agar plates to obtain a pure bacterial isolate. Additional tests were performed to determine the species of the isolated pathogens including catalase, oxidase, bile solubility, optochin, X/V growth factor, and/or API NH strips [17]. If the isolate was identified as *N. meningitidis* or *H. influenzae*, slide agglutination serogrouping or slide agglutination serotyping, respectively, was performed [7] to identify *N. meningitidis* serogroups and Hib. The NRL characterized *S. pneumoniae* only at the species level. The NRL did not test for other *H. influenzae* serotypes, therefore only Hib and *H. influenzae* non-b results were reported. Beginning in 2016, the NRL also performed direct rt-PCR on CSF, isolates, and trans-isolate media inoculated with CSF [7, 17, 18] using Quanta PerfeCTa ToughMix Low ROX master mix (QuantaBio) and primer-probe sets targeting specific genes for species identification or speciation (*sodC* for *N. meningitidis*, *hpd* for *H. influenzae*, and *lytA* for *S. pneumoniae*), capsular genogrouping of *N. meningitidis* (*csaB* for NmA, *csb* for NmB, *csc* for NmC, *csw* for NmW, *csxB* for NmX, and *csy* for NmY), and capsular genotyping of *H. influenzae* (*bcs3* for Hib) [17, 18].

If 1 of the 3 pathogens is detected, the final interpretation is recorded by the NRL as positive for bacterial meningitis even if other organisms that are not associated with bacterial meningitis are also detected. If the test results are negative or other pathogen (only 1 nonmeningitis pathogen isolated from a specimen), the interpretation is recorded as negative for the 3 bacterial meningitis pathogens (*N. meningitidis*, *H. influenzae*, and *S. pneumoniae*). Contaminated (multiple pathogens cultured with no isolated bacterial meningitis pathogens) or undetermined (test results in the equivocal range, which is neither positive nor negative) results are recorded as inconclusive. Finally, if tests are not performed, in progress, or no information is provided, the interpretation is recorded as no data.

To report a final case determination, available results from the confirmatory tests (culture, latex, and rt-PCR) were assessed using the following criteria. If the results of all 3 confirmatory tests were congruent, this result was reported as the final interpretation. If the test results were discordant, the rt-PCR result was reported as final unless the rt-PCR result was negative, inconclusive, or no data. In this case, the culture result was reported as the final interpretation unless the culture result was reported as negative, inconclusive, or no data. Otherwise, the latex result was used for the final case determination. If none of the confirmatory testing methods were performed, the final interpretation was recorded as no data.

We describe analysis of meningitis specimens at the NRL during 3 periods: pre-MACV (2010–2012), pre-MenAfriNet (2013–2015), and post-MenAfriNet (2016–2018). We calculated the proportion of suspected cases with a specimen received by period, and the proportion of specimens with a bacterial meningitis pathogen identified by period, pathogen, and test.

The 95% confidence intervals (CIs) were calculated using the exact binomial method for the following result categories: the number of specimens testing positive, negative, inconclusive, and with no data. Additionally, 95% CIs were calculated for the laboratory results of specimens tested by both rt-PCR and culture-based methods in 2016–2018.

## RESULTS

**Figure 1.**
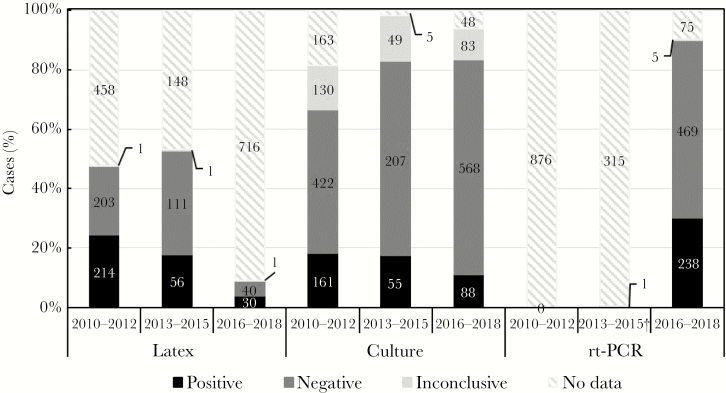
Specimens received and tested by 3 confirmatory methods in Chad,

During 2010–2012, 12,813 suspected cases were aggregately reported nationwide; 876 (6.8%) specimens were received and 721 (82.3%) tested at the NRL. From 2013 to 2015, 681 suspected meningitis cases were reported; 316 (46.4%) specimens were received and 313 (99.1%) tested at the NRL. From 2016 to 2018, Chad reported 995 suspected meningitis cases; 787 (79.1%) specimens were received and 773 (98.2%) were tested at the NRL (Table 1). Of the 876 specimens received at the NRL during 2010–2012, 418 (47.7%) and 713 (81.4%) were tested by latex and culture, respectively (Figure 1). For 2013–2015, 168 (53.2%) and 311 (98.4%) of the 316 specimens received were tested by latex and culture, respectively. After MenAfriNet implementation (2016–2018), 71 (9.0%), 739 (93.9%), and 712 (90.5%) of the 787 specimens received were tested by latex, culture, and rt-PCR, respectively. Additionally, 678 (86.1%) specimens were tested by both culture and rt-PCR during 2016–2018 (Table 2). Of the 678 cases, 85 (12.5%) were identified as positive by culture and 220 (32.4%) by rt-PCR. Overall, of the specimens received, the NRL confirmed 33.6% (2010–2012), 27.8% (2013–2015), and 33.2% (2016–2018) as positive for 1 of the 3 bacterial meningitis pathogens (Table 1). The percentage of specimens testing negative were 36.1% (2010–2012), 56.0% (2013–2015), and 57.6% (2016–2018). The remaining specimens yielded either inconclusive results or no data (Table 1).

**Table 1.** Suspected Meningitis Cases, Received Specimens, and Case Confirmation Results in Chad, 2010–2018

Year	No. of Suspected Cases	No. of Specimens Received, (%)^a^	No. of Specimens Positive for Nm, Hi, or Sp (%; 95% CI)^b^	No. Specimens Negative for Nm, Hi, or Sp (%; 95% CI)^b^	No. of Specimens Inconclusive,^c^ (%, 95% CI)^b^	No. of Specimens with no data,^d^ (%, 95% CI)^b^
Pre-MACV	12813	876 (6.8)	294 (33.6; 30.5–36.8)	316 (36.1; 32.9–39.4)	111 (12.7; 10.6–15.1)	155 (17.7; 15.2–20.4)
2010	3058	143 (4.7)	45 (31.5; 24.0–39.8)	71 (49.7; 41.2–58.2)	15 (10.5; 6.0–16.7)	12 (8.4; 4.4–14.2)
2011	5960	391 (6.6)	107 (27.4; 23.0–32.1)	139 (35.5; 30.8–40.5)	33 (8.4; 5.8–11.6)	112 (28.6; 24.2–33.4)
2012	3795	342 (9.0)	142 (41.5; 36.2–46.9)	106 (31.0; 26.1–36.2)	63 (18.4; 14.4–22.9)	31 (9.1; 6.3–12.7)
Post-MACV						
Pre-MenAfriNet	681	316 (46.4)	88 (27.8; 22.9–33.1)	177 (56.0; 50.3–61.6)	48 (15.2; 11.4–19.6)	3 (0.9; .2–2.7)
2013	242	141 (58.3)	42 (29.8; 22.4–38.1)	69 (48.9; 40.4–57.5)	28 (19.9; 13.7–27.4)	2 (1.4; .2–5.0)
2014	214	96 (44.9)	28 (29.2; 20.4–39.4)	55 (57.3; 46.8–67.3)	12 (12.5; 6.6–20.8)	1 (1.0; .0–5.6)
2015	225	79 (35.1)	18 (22.8; 14.1–33.6)	53 (67.1; 55.6–77.5)	8 (10.1; 4.4–19.0)	0 (0.0; .0–4.6)
Post-MenAfriNet^e^	995	787 (79.1)	261 (33.2; 29.9–36.6)	453 (57.6; 54.1–61.1)	59 (7.5; 5.8–9.6)	14 (1.8; 1.0–3.0)
2016	206	114 (55.3)	38 (33.3; 24.7–42.7)	65 (57.0; 47.4–66.2)	9 (7.9; 3.7–14.5)	2 (1.8; .3–6.2)
2017	454	396 (87.2)	121 (30.6; 26.1–35.4)	242 (61.1; 56.1–65.9)	31 (7.8; 5.4–10.9)	2 (0.5; .1–1.8)
2018^f^	335	277 (82.7)	102 (36.8; 31.1–42.8)	146 (52.7; 46.6–58.7)	19 (6.9; 4.2–10.5)	10 (3.6; 1.7–6.5)

Abbreviations: CI, confidence interval; Hi, *Haemophilus influenzae*; Nm, *Neisseria meningitidis;* MACV, meningococcal serogroup A conjugate vaccine; MenAfriNet, Meningitis Africa Network; Sp, *Streptococcus pneumoniae.*

^a^Percentages are calculated using the number of suspected cases as the denominator.

^b^Percentages are calculated using the number of specimens received as the denominator.

^c^Inconclusive results include the final interpretations contaminated and undetermined. Contamination is the final interpretation when the cultured specimen is too contaminated to isolate bacterial meningitis pathogens.

^d^If a test was not performed, the results were categorized as no data, which include in progress, not tested, or no information given.

^e^MenAfriNet was not introduced in Chad until April 2016.

^f^Weeks 1–28.

**Table 2.** Laboratory Results of Specimens Tested by Both rt-PCR and Culture, 2016–2019 (n = 678)

Result	No. Tested Positive by PCR (%; 95% CI)	No. Tested Positive by Culture (%; 95%CI)
Nm	66 (9.7; 7.6–12.2)	19 (2.8; 1.7–4.3)
NmW	15 (2.2; 1.2–3.6)	3 (0.4; .1–1.3)
NmX	45 (6.6; 4.8–8.7)	13 (1.9; 1.0–3.2)
Other Nm	6 (0.9; .3–1.9)	3 (0.4; .1–1.3)
Hi^a^	34 (5.0; 3.5–6.9)	14 (2.1; 1.1–3.5)
Sp	120 (17.7; 14.9–20.8)	52 (7.7; 5.8–10.0)
Total confirmed	220 (32.4; 28.9–36.1)	85 (12.5; 10.1–15.2)
Negative	453 (66.8; 63.1–70.3)	511 (75.4; 72.0–78.6)
Inconclusive	5 (0.7; .2–1.7)	82 (12.1; 9.7–14.8)

Abbreviations: CI, confidence interval; Hi, *Haemophilus influenzae*; NmW, *Neisseria meningitidis* serogroup W; NmX, *Neisseria meningitidis* serogroup X; Other Nm, other *Neisseria meningitidis* groups, which are nongroupable, polyagglutinate, and autoagglutinate; rt-PCR, real-time polymerase chain reaction; Sp, *Streptococcus pneumoniae.*

^a^All of the identified Hi were Hib.

2010–2012 (n = 876), 2013–2015 (n = 316), and 2016–2018 (n = 787). If a test was not performed, the results were categorized as no data, which included in progress, not tested, or no information given. Inconclusive test results were contaminated or undetermined. ^†^One case was collected in 2013. This case was tested and confirmed in 2017 using real-time polymerase chain reaction.

Of the confirmed bacterial meningitis cases identified in 2010–2012, 73.5% were tested by latex with 98.6% positive, whereas 97.6% were tested by culture with 56.1% positive. During 2013–2015, 75.0% of the confirmed cases were tested by latex with 80.3% positive. By culture, 98.9% of confirmed cases were tested with 63.2% positive. During 2016–2018, 14.6%, 93.1%, and 98.9% of confirmed cases were tested by latex, culture, and rt-PCR, respectively, with 63.2%, 34.6%, and 92.2% confirmed by each method, correspondingly (Table 3).

**Table 3.** Pathogen Detection by 3 Testing Methods, Chad

		Latex		Culture		rt-PCR^d^	
Result	Total No. of Confirmed Pathogens^a^	No. Tested^b^ (%)	No. Positive^c^ (%)	No. Tested (%)	No. Positive (%)	No. Tested (%)	No. Positive (%)
2010–2012							
Nm	278	206 (74.1)	204 (99.0)	274 (98.6)	151 (55.1)	…	…
NmA	254	184 (72.4)	183 (99.5)	250 (98.4)	145 (58.0)	…	…
NmW^e^	20	20 (100.0)	19 (95.0)	20 (100.0)	4 (20.0)	…	…
NmX^f^	2	0 (0.0)	0 (0.0)	2 (100.0)	2 (100.0)	…	…
Other Nm	2	2 (100.0)	2 (100.0)	2 (100.0)	0 (0.0)	…	…
Hi^g^	1	1 (100.0)	1 (100.0)	1 (100.0)	0 (0.0)	…	…
Sp	15	9 (60.0)	8 (88.9)	12 (80.0)	10 (83.3)	…	…
Total confirmed	294	216 (73.5)	213 (98.6)	287 (97.6)	161 (56.1)	…	…
2013–2015							
Nm^h^	20	18 (90.0)	17 (94.4)	20 (100.0)	6 (30.0)	…	…
NmA	2	1 (50.0)	1 (100.0)	2 (100.0)	1 (50.0)	…	…
NmW^e^	16	16 (100.0)	15 (93.8)	16 (100.0)	5 (31.3)	…	…
NmX^f,i^	1	0 (0.0)	0 (0.0)	1 (100.0)	0 (0.0)	…	…
Other Nm	1	1 (100.0)	1 (100.0)	1 (100.0)	0 (0.0)	…	…
Hi^g^	10	8 (80.0)	5 (62.5)	10 (100.0)	7 (70.0)	…	…
Sp	58	40 (69.0)	31 (77.5)	57 (98.3)	42 (73.7)	…	…
Total confirmed	88	66 (75.0)	53 (80.3)	87 (98.9)	55 (63.2)	…	…
2016–2018							
Nm	86	14 (16.3)	7 (50.0)	73 (84.9)	17 (23.3)	85 (98.8)	79 (92.9)
NmA	0	0 (0.0)	0 (0.0)	0 (0.0)	0 (0.0)	0 (0.0)	0 (0.0)
NmW^e^	17	4 (23.5)	4 (100.0)	16 (94.1)	2 (12.5)	17 (100.0)	16 (94.1)
NmX^f^	58	6 (10.3)	0 (0.0)	46 (79.3)	13 (28.3)	58 (100.0)	57 (98.3)
Other Nm	10	3 (30.0)	2 (66.7)	10 (100.0)	2 (20.0)	9 (90.0)	6 (66.7)
Hi^g^	39	6 (15.4)	5 (83.3)	38 (97.4)	15 (39.5)	38 (97.4)	35 (92.1)
Sp	136	18 (13.2)	12 (66.7)	132 (97.1)	52 (39.4)	135 (99.3)	124 (91.9)
Total confirmed	261	38 (14.6)	24 (63.2)	243 (93.1)	84 (34.6)	258 (98.9)	238 (92.2)

Abbreviations: Hi, *Haemophilus influenzae*; NmA, *Neisseria meningitidis* serogroup A; NmW, *Neisseria meningitidis* serogroup W; NmX, *Neisseria meningitidis* serogroup X; Other Nm, other *Neisseria meningitidis* groups, which are nongroupable, polyagglutinate, and autoagglutinate; rt-PCR, real-time polymerase chain reaction; Sp, *Streptococcus pneumoniae.*

^a^Distribution of pathogens is from the final case interpretations, which use the results from all test methods. The definition of final case interpretation is included in the “Methods” section.

^b^Percentage of specimens tested is calculated using the total number of pathogens (second column) as the denominator.

^c^The percentage of positive cases is calculated using the number of specimens tested (third column) as the denominator.

^d^rt-PCR was not implemented prior to 2016.

^e^Test results are reported as NmW/Y for latex because the test cannot differentiate between NmW and NmY.

^f^Latex does not test for NmX.

^g^All of the identified Hi were Hib. Hi non-b has not been detected during these time periods.

^h^One specimen was positive for NmB/*Escherichia coli* by latex in 2017 (post-MenAfriNet). The specimen tested negative for Nm, Hib, and Sp by culture and rt-PCR, and is excluded from the table.

^i^One NmX case, collected in 2013, was tested and confirmed in 2017 using rt-PCR.

**Figure 2.**
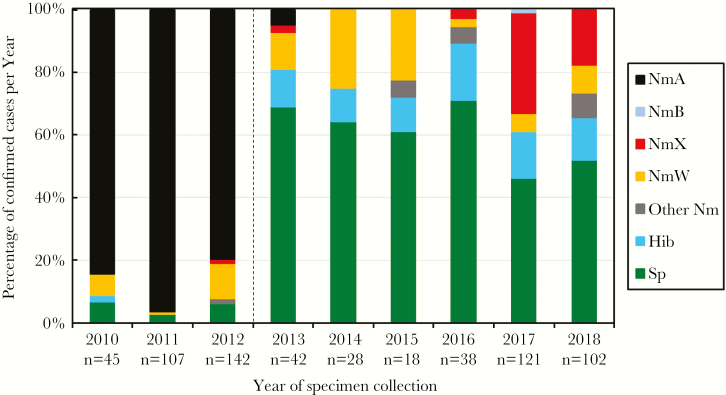
Epidemiology of bacterial meningitis in Chad, 2010–2018. Dotted line indicates MACV vaccination campaigns conducted in Chad from 2011 to the end of 2012. Abbreviations: Hib, *Haemophilus influenzae* serotype b; NmA, *Neisseria meningitidis* serogroup A; NmB, *N. meningitidis* serogroup B; NmX, *N. meningitidis* serogroup X; NmW, *N. meningitidis* serogroup W; Other Nm, other *N. meningitidis*, which are nongroupable, polyagglutinate, and autoagglutinate; Sp, *Streptococcus pneumoniae.*

From 2010 to 2012, the majority of confirmed bacterial meningitis cases were NmA (86.4%) (Figure 2). By 2013, 1 year after the MACV mass vaccination campaign completion, 2 NmA cases were detected, accounting for 4.8% of confirmed cases (Table 3 and Figure 2). Since 2014, no NmA cases have been identified (Table 3 and Figure 2). However, there was an increase in both absolute number and percentage of confirmed cases caused by other species and *N. meningitidis* serogroups. Among confirmed bacterial meningitis cases, 5.1% were *S. pneumoniae* during 2010–2012, 65.9% during 2013–2015, and 52.1% during 2016–2018. The percentage of cases confirmed as NmX were 0.7% during 2010–2012, 1.1% during 2013–2015, and 22.2% during 2016–2018. Similarly, Hib represented 0.3% of confirmed cases during 2010–2012, 11.4% during 2013–2015, and 14.9% during 2016–2018. There were zero *H. influenzae* non-b reported.

## DISCUSSION

Analysis of surveillance data from 2010–2018 revealed a shift in the distribution of bacterial meningitis pathogens identified through testing at the NRL. Prior to the MACV campaign in Chad, NmA was the predominant pathogen. However, following the completion of MACV campaigns in 2012, there was a substantial reduction in NmA cases, and non-NmA pathogens were identified from the majority of confirmed bacterial meningitis cases. Specifically, *S. pneumoniae*, NmX, and Hib have become the dominant pathogens identified. The pneumococcal vaccine (PCV) is not yet a part of Chad’s routine vaccination schedule [19]. Although the Hib vaccine has been included in the routine schedule since 2008, as of 2017, its coverage in Chad is less than 50% [20]. Currently, there are no reports that indicate a plan to introduce PCV or improve Hib coverage in Chad.

The reduction in non-NmA cases is a pattern that has also been observed in other meningitis belt countries post-MACV [3, 21, 22]. Since its introduction, meningococcal meningitis in the meningitis belt has largely been attributed to NmC and NmW [3]. As an example, Burkina Faso was the first country to implement a MACV campaign in 2010, and the majority of cases reported after introduction (2011–2015) were confirmed as *S. pneumoniae* (57%) and *N. meningitidis* (40%), with NmW as the predominant serogroup (64%) [21]. In Chad, however, there has been an increase in the number of NmX cases. The observed increase in non-NmA cases may be due to both the implementation of CBS and improved detection by rt-PCR, particularly for NmX, as latex (Pastorex) is not capable of identifying this pathogen. An overall increase in disease incidence could also be a contributing factor. One important limitation of this study is that CBS data were not available for all districts in the country post-MACV, specifically 2014–2018, and during the pre-MACV era select districts operated under CBS for a limited period of time. Consequently, the epidemiology of bacterial meningitis for these time periods may be incomplete.

The lower percentage of specimens received at the NRL during 2010–2012 (6.8%), compared to 2013–2015 (46.4%), is not surprising given that Chad was only operating under EMS (2010–2011) before CBS was implemented in a few districts in 2012. The lower percentage of specimen received during 2010–2012 may also be due to a higher number of suspected cases reported and lower lumber puncture rate during the large NmA outbreak [3]. The progressive introduction of CBS to additional districts (supported by MenAfriCar during 2012–2013, MSF starting in 2012, and MenAfriNet since 2016) likely contributed to the increase in the proportion of specimens received during 2016–2018 (79.1%) compared to 46.4% during 2013–2015. However, in the absence of information on specimen handling practices for each suspected case of meningitis prior to MenAfriNet-supported CBS implementation, it cannot be determined whether the observed increase post-MenAfriNet implementation was due to improved specimen collection and/or improved specimen transport practices. Of the specimens received and tested at the NRL during each time period, a notable number of cases were negative or inconclusive for the 3 bacterial meningitis pathogens. Possible factors contributing to the higher number of cases with negative or inconclusive test results include clinical interpretation of the suspected meningitis case definitions and/or causative pathogens other than *N. meningitidis*, *H. influenzae*, and *S. pneumoniae*. Similarly, suboptimal storage/transport conditions, specimen accessioning and processing practices, and specimen handling habits (eg, multiple freeze-thaw cycles) can negatively affect specimen quality and cause inconclusive or negative results when tested by latex, culture, and/or rt-PCR.

Prior to implementation of rt-PCR in 2016, latex and culture methods were predominantly used by Chad’s NRL to identify bacterial meningitis pathogens. Latex detects bacterial capsular antigens for the identification of the *N. meningitidis* serogroups and *H. influenzae* serotypes [10], while culture identification is dependent on the recovery of a viable isolate from a specimen [8]. In contrast, rt-PCR targets specific genes and does not require a viable pathogen [18]. The rt-PCR method offers several other advantages, including the provision of results within hours, high throughput, and high sensitivity and specificity [18, 23]. Latex was commonly performed at the peripheral level (district hospital and health center laboratories) as a rapid diagnostic test (result in less than 20 minutes); however, the commercially available test (Pastorex) does not detect NmX, nor does it distinguish NmW from NmY, or NmB from *Escherichia coli* [24]. Notably, 50% of the NmX cases tested by latex during 2016–2018 were misidentified as NmW but confirmed as NmX by rt-PCR (data not shown). Following rt-PCR implementation, Chad has reduced the use of latex for a number of reasons, including high acquisition and distribution costs, requirement for cold storage, short shelf-life, and the need for extensive training. Unlike latex and rt-PCR, culture requires 16 hours or longer to obtain a final result. Additionally, recovering a viable isolate is often difficult in resource-limited settings where specimen transport and storage conditions may negatively affect specimen quality. Despite this challenge, culture remains the standard method for bacterial meningitis diagnosis [25]. Therefore, rt-PCR and culture are now the primary confirmatory testing methods used for bacterial meningitis surveillance in Chad.

## CONCLUSION

In this report, we demonstrate that specimen receipt and pathogen detection at the NRL in Chad increased following CBS and rt-PCR implementation. A shift in the distribution of bacterial meningitis pathogens detected was observed, with pneumococcus as the predominant cause of bacterial meningitis post-MACV and an increase in the number of reported cases due to NmX and Hib. Continued surveillance with laboratory confirmation using sensitive and specific tests, such as rt-PCR, to monitor the changing epidemiology of meningitis can inform the development of effective vaccination strategies.

## Notes

***Acknowledgments.*** We acknowledge contributions to this work by members of the Hôpital Général de Référence National Laboratory, the MenAfriNet Consortium, MenAfriCar consortium, Epicentre (Paris, France), Médecin Sans Frontières, and the Bacterial Meningitis Laboratory, Centers for Disease Control and Prevention (CDC). Writing support was provided by Mary Ann Hall.

***Financial support*.** This work was funded by the MenAfriNet consortium (www.menafrinet.org) through a grant from the Bill & Melinda Gates Foundation (OPP1084298).

***Potential conflicts of interest.*** All authors: No reported conflicts of interest. All authors have submitted the ICMJE Form for Disclosure of Potential Conflicts of Interest. Conflicts that the editors consider relevant to the content of the manuscript have been disclosed.
